# Efficacy and safety of mycophenolate mofetil in patients with IgA nephropathy: an update meta-analysis

**DOI:** 10.1186/s12882-017-0647-x

**Published:** 2017-07-19

**Authors:** Bing Du, Ye Jia, Wenhua Zhou, Xu Min, Lining Miao, Wenpeng Cui

**Affiliations:** 1 Department of Cardiology, the Second Part of First Hospital, Jilin University, Changchun, 130031 China; 2 Department of Nephrology, Second Hospital, Jilin University, 218 Ziqiang Street, Changchun, Jilin, 130041 China

**Keywords:** Immunoglobulin a nephropathy, Meta-analysis, Mycophenolate mofetil

## Abstract

**Background:**

The application of mycophenolate mofetil (MMF) in treating patients with immunoglobulin A nephropathy (IgAN) remains uncertain. This update meta-analysis was performed to re-evaluate the therapeutic potential of MMF in IgAN.

**Methods:**

Articles were obtained by searching the electronic databases without language restriction. Randomized controlled trials studying the role of MMF in treating IgAN were collected. The quality of included studies was critically evaluated. Data analyses were performed by using RevMan 5.3 software.

**Results:**

A total of 297 articles were screened and eight articles were finally included. Among the eight randomized controlled trials, five and three were high quality and low quality, respectively. Both fixed-effect and random-effect model were used. Pooled results by combining all the eight studies suggested that IgAN patients in MMF group had a higher remission rate than that in control group. Compared to placebo or corticosteroid monotherapy, MMF monotherapy exerted a higher remission rate and side effect rate in both main analysis and subgroup analysis by human race. Compared to corticosteroid plus other immunosuppressive agent therapy, corticosteroid plus MMF therapy had a higher remission rate, lower serum creatinine doubling rate, progression to end-stage renal disease rate and side effects rate. Subgroup analysis by therapeutic regimen further confirmed these results between corticosteroid plus MMF therapy and corticosteroid plus cyclophosphamide therapy. Funnel-plot displayed a symmetrical figure, indicating no publication bias existed.

**Conclusions:**

MMF has the potential in treatment of IgAN, especially for Asians. The evidence currently available shows that MMF monotherapy has a more efficacy but higher side effects when compared to placebo or corticosteroid monotherapy in treatment of Asians with IgAN. While MMF combined with corticosteroid regimen has a more efficacy and lower side effects when compared with corticosteroid plus cyclophosphamide regimen.

## Background

Immunoglobulin A nephropathy (IgAN) is the most common form of primary glomerulonephritis worldwide and is the leading cause of end-stage renal disease (ESRD) [1, 2]. According to Kidney Disease Improving Global outcomes (KDIGO) guideline, renin-angiotensin system inhibitor for IgAN patients with persistent proteinuria ≥0.5 g/d, and renin-angiotensin system inhibitor plus corticosteroid for IgAN patients with persistent proteinuria ≥1 g/d are recommended [3]. However, up to 30% of the treated patients fail to respond to these therapies [4, 5]. Therefore the lack of satisfactory therapeutic approach for IgAN still confuses physicians and researchers working in nephrology. The predominant character of IgAN is abnormal IgA1 deposition in mesangial area. Moreover, molecular and cellular interaction studies [6], as well as genome-wide association studies [7] revealed the autoimmune nature of this disease. These knowledges provide nephrologists a theoretical basis for the treatment of IgAN with immunosuppressive therapy.

Mycophenolate mofetil (MMF), a highly effective immunosuppressive agent, acts by releasing mycophenolic acid which leads to apoptosis of cytotoxic T-lymphocytes and reduction of antibody synthesis via selectively inhibits T- and B-lymphocyte proliferation [8, 9]. In addition, growing clinical evidences have demonstrated that oral MMF is beneficial for IgAN secondary to systemic diseases, such as lupus nephritis [10] and Henoch-Schonlein purpura nephritis [11]. However, the application of MMF in treatment patients with primary IgAN is still uncertain. So far, few randomized controlled trails (RCTs) have studied the therapeutic effects of MMF on IgAN. The first RCT investigating the role of MMF in patients with IgAN was carried out by Chen et al. in 2002 [12]. This study demonstrated that compared to prednisone, MMF was more effective in reducing proteinuria in patients with severe IgAN [12]. Another RCT from China also claimed that corticosteroid-free MMF monotherapy was effective in decreasing proteinuria and ameliorating some of the abnormalities in IgAN [13]. Moreover, a six-year follow-up of the same cohort also suggested a kidney survival benefit in patients treated with MMF monotherapy [14]. In contrast to studies from Asians [12–14], in a prospective placebo-controlled randomized study carried out in Belgium, patients were treated with restriction of salt intake, angiotensin converting enzyme inhibitors and either MMF or placebo [15]. After 36 months of follow-up, however, no beneficial effects of MMF treatment could be demonstrated on renal function or proteinuria [15]. One year later, similar result was reported in another double-blind, randomized, placebo-controlled trial from USA [16]. Because of the inconsistency between these RCTs mentioned above, MMF was not recommended in treating IgAN by KDIGO guideline in 2012 [3].

So far, more RCTs have provided the evidence for the effectiveness of MMF therapy in IgAN [17–19]. Therefore re-evaluating the usage of MMF in treating patients with IgAN seems to be necessary. In a recent meta-analysis, both efficacy and safety of MMF regimen in treating IgAN were estimated [20]. However, there were some limitations in this study. First, one trial included in this meta-analysis contained obviously incorrect data [21]. Second, subgroup analysis by human race was not taken.

Considering these limitations may lead to unreliable conclusion, we carried out this update meta-analysis to comprehensively re-evaluate the efficacy and safety of MMF therapy in treating IgAN. In current meta-analysis, we added one new published RCT [19], and removed one study with obviously incorrect data [21].

## Methods

### Search strategy

Our study protocol and analysis were planned in accordance with the Preferred Reporting Items for Systematic Reviews and Meta-Analyses (PRISMA) guidelines [22]. Eligible studies were obtained by systematically searching the electronic databases of EMBASE, MEDLINE, the Cochrane Library, and China National Knowledge Infrastructure without language restriction. In addition, the following key words and subject terms were used in the search strategy: Berger’s disease, immunoglobulin A nephropathy, IgA nephropathy, IgAN, IgA nephritis, IgA glomerulonephritis, mycophenolate mofetil, MMF, mycophenolic acid, and their derivative words. All studies, published up to December 2015, focusing on the efficacy and safety of MMF in IgAN were considered to be included in our meta-analysis. Moreover, we also took out a manual search of abstracts from selected conferences. No ethical approval and patient consent are required, because the current study is based on previous published studies.

### Inclusion and exclusion criteria

Two authors (Du B and Min X) conducted the literature search and selection independently. Discrepancies were resolved by consultation and discussion with the third authors (Cui W). The title and abstract of potential studies were screened for appropriateness before full article intensive reading. Inclusion criteria: (a) all cases were renal biopsy-proven IgAN, (b) the study design was RCT, and (c) the efficacy in treating IgAN was compared between MMF monotherapy and placebo or between MMF monotherapy and corticosteroid monotherapy or between MMF and other immunosuppressive agents on the basis of corticosteroid. Exclusion criteria: (a) the quality of study was too low, (b) the study was just a trial protocol, and (c) the study did not clearly report the primary outcome (remission rate).

### Data extraction

For each included study, the following information was extracted separately by two authors (Du B and Min X): first author, year of publication, study design, human race of the participants, simple size, treatment proposal, time of follow-up, primary outcome (remission rate), secondary outcomes (urinary protein reduction, serum creatinine doubling rate and progression to ESRD rate) and adverse events.

### Study quality assessment

Jadad score was used to assess the methodologic quality of the included trials by two authors (Jia Y and Zhou W). Studies gained 1–2 points were regarded as low quality, while the ones gained 3–5 points were regards as high quality [23].

### Statistical methods

The primary outcome was remission rate and secondary outcomes included reduction of proteinuria, serum creatinine doubling rate and progression to ESRD rate. For dichotomous data, such as remission rate, serum creatinine doubling rate, progression to ESRD rate and side effect rate, pooled odds ratio (OR) with the corresponding 95% confidence interval (CI) was used. For continuous data, such as urinary protein reduction, weighted mean difference (WMD) was used. ORs or WMD of different RCTs were combined by using the random-effects model if true between-study heterogeneity existed or else using the fixed-effects model instead.

Both I^2^ and Q statistics were considered for testing heterogeneity between studies [24]. The I^2^ takes values between 0 and 100%, with higher values denoting greater degree of heterogeneity (I^2^ = 0–25%, 25%–50%, 50%–75% and 75%–100% represents no, moderate, large and extreme heterogeneity, respectively). What’s more, we also performed subgroup analysis to explore underlying sources of heterogeneity. Visual analysis of the funnel plot was made to assess the publication bias. The statistical software packages for managing and analyzing all aspects of a Cochrane Collaboration systematic review, Review Manager 5.3, was used in current study.

## Results

### Characteristics of trials

**Fig. 1:**
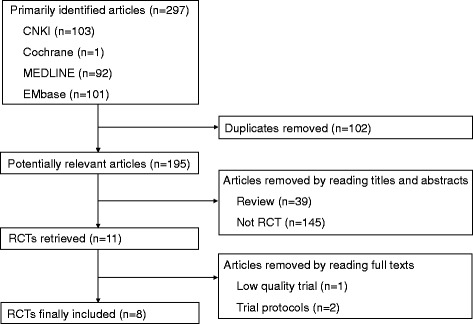
Flow diagram of study selection. The number of citations retrieved by individual searches, the final number of RCTs included in the meta-analysis, and reasons for exclusions are provided. RCT, randomized controlled trial

There were 297 articles relevant to the search term and eight articles [12, 13, 15–19, 25] involving 347 patients with IgAN (MMF group: 178 patients, control group: 169 patients) were included in this meta-analysis finally. Of the eight studies, there were five and three studies using corticosteroid-free, MMF monotherapy [12, 13, 15, 16, 19] and corticosteroid plus MMF therapy [17, 18, 25], respectively. Moreover, there were five, two and one RCTs investigating Asians [12, 13, 17, 18, 25], Caucasians [15, 16] and mixed races [19], respectively. The flow chart for the selection of RCTs to be included in our analysis was shown in Fig. 1. The characteristics of these trials were showed in Table 1.

**Table 1:** Basic characteristics of included studies

Studies	Racial decent (country)	Study design	Therapeutic regimen	Sample size	Time of follow-up (months)	Random method	Withdraw & lost to follow-up	Blinding	Jadad Score
Hogg 2015 [19]	Mixed (Canada)	RCT	MMF (25–36 mg/kg, max 2.0 g/day)	22	6	1	1	1	3
			Placebo	22					
Liu 2014 [18]	Asians (China)	RCT	MMF (1.5 g/day) + prednisone	42	12	2	1	0	3
			CTX + prednisone	42					
Liu 2010 [17]	Asians (China)	RCT	MMF (1.5 g/day) + prednisone	20	6	1	1	0	2
			LEF + prednisone	20					
Bao 2007	Asians (China)	RCT	MMF (2.0 g/day) + prednisone	18	12	1	1	0	2
			CTX + prednisone	16					
Frich 2005	Caucasians (America)	RCT	MMF (2.0 g/day)	17	24	2	1	2	5
			Placebo	15					
Tang 2005 [13]	Asians (China)	RCT	MMF (2.0 g/day)	20	18	1	1	1	3
			Placebo	20					
Baes 2004	Caucasians (Belgium)	RCT	MMF (2.0 g/day)	21	36	1	1	1	3
			Placebo	13					
Chen 2002 [12]	Asians (China)	RCT	MMF (1.5 g/day)	18	18	1	1	0	2
			prednisone	21					

### Methodologic quality assessment

All the trials included in this meta-analysis mentioned the term “random”, but the detail method was illuminated in two articles only [16, 18]. There were four trials mentioned the term “double blind” [13, 16, 19, 25], but only one article explained the detail method [16]. All the eight trials described the data of the patients who withdrew during the treatment period. According to the Jadad score, five and three articles were regarded as high quality literatures [13, 15, 16, 18, 19] and low quality literatures [12, 17, 25], respectively (Table 1).

### Heterogeneity test

**Fig. 2:**
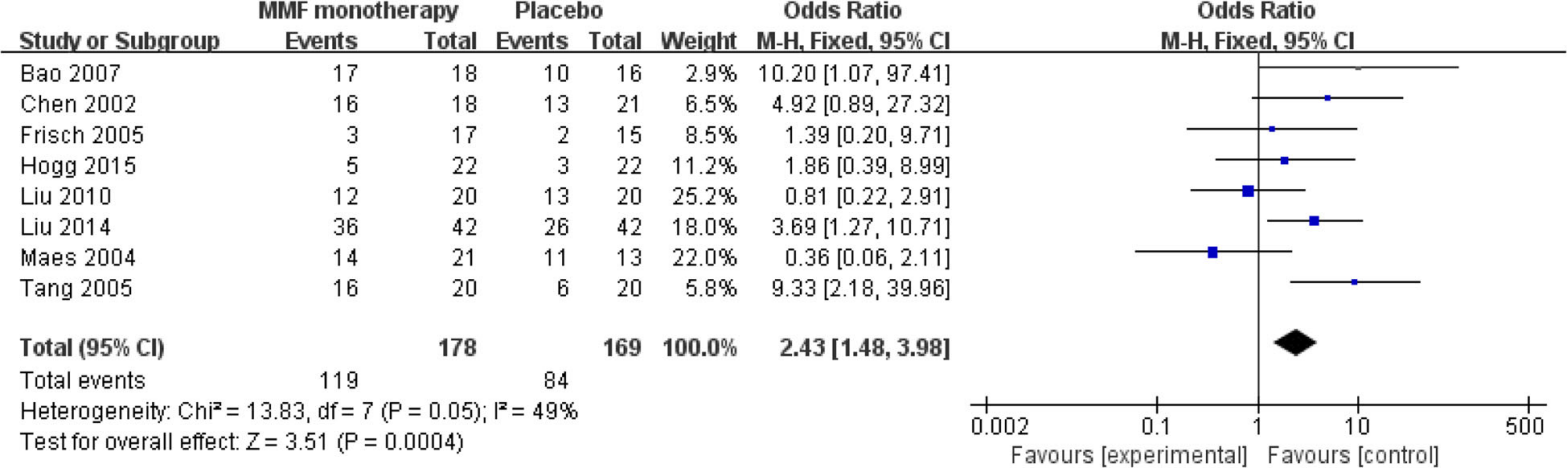
Forest plot of remission rate of IgAN patients treated with MMF or other therapy. IgAN, immunoglobulin A nephropathy; MMF, mycophenolate mofetil

For all including studies, fixed-effect model was chosen to combine the results because no significant heterogeneities between studies in analyses for remission rate (Fig. 2).

**Fig. 3:**
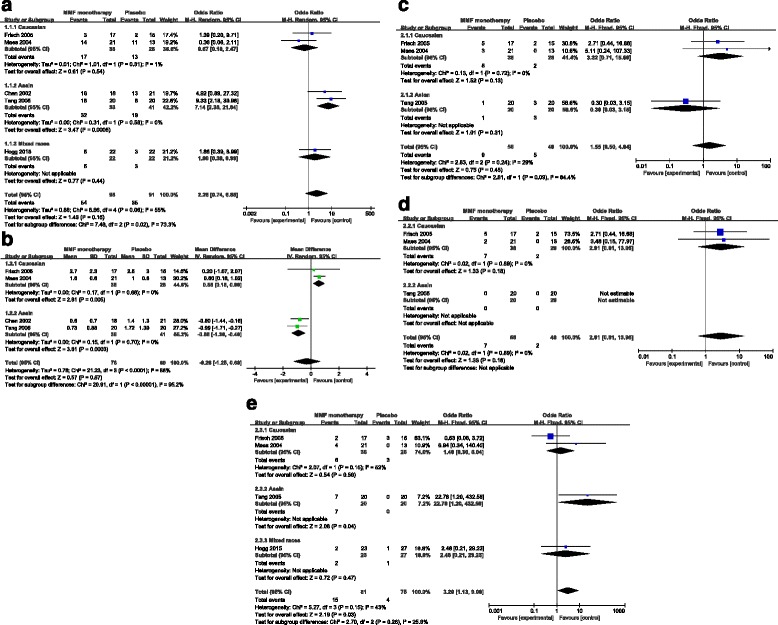
Forest plot of outcomes of IgAN patients treated with MMF monotherapy or corticosteroid monotherapy or placebo. Main analysis and subgroup analysis by human race of remission rate (**a**), urinary protein reduction (**b**), serum creatinine doubling rate (**c**), progression to ESRD rate (**d**) and side effect rate (**e**) are provided. ESRD, end-stage renal disease; IgAN, immunoglobulin A nephropathy; MMF, mycophenolate mofetil

For studies using MMF monotherapy, random-effect model was chosen to combine the results because there were significant heterogeneities between studies in analyses for urinary protein reduction rate. Fixed-effect model was chosen to combine the results because no significant heterogeneities between studies in analyses for remission rate, serum creatinine doubling rate, progression to ESRD rate and side effect rate were found (Fig. 3).

**Fig. 4:**
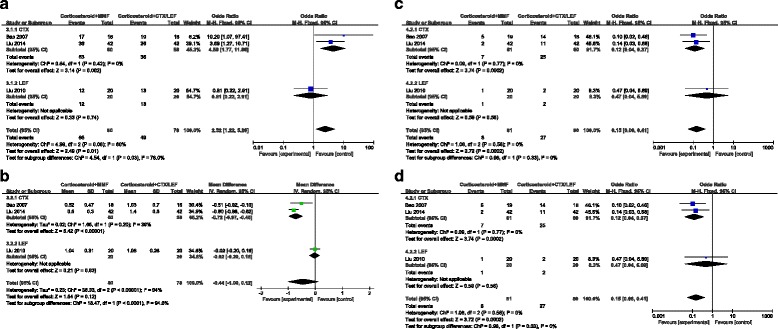
Forest plot of outcomes of IgAN patients treated with corticosteroid plus MMF therapy or corticosteroid plus other immunosuppressive agents. Main analysis and subgroup analysis by therapeutic regimen of remission rate (**a**), urinary protein reduction (**b**), serum creatinine doubling rate (**c**) and side effect rate (**d**) are provided. CTX, cyclophosphamide; IgAN, immunoglobulin A nephropathy; LEF, leflunomide; MMF, mycophenolate mofetil

For studies using corticosteroid plus MMF therapy, random-effect model was chosen to combine the results because there were significant heterogeneities between studies in analyses for urinary protein reduction rate. Fixed-effect model was chosen to combine the results because no significant heterogeneities between studies in analyses for remission rate, serum creatinine doubling rate and side effect rate were found (Fig. 4).

### Pooled results of all including studies

Remission rate was recorded in all these eight trials. The main analysis revealed that the remission rate in MMF group was significant higher than that in control group (Z = 3.51, *P* = 0.0004) (Fig. 2).

### Pooled results of studies using MMF monotherapy

Five studies evaluated the role of MMF monotherapy in treatment of IgAN patients [12, 13, 15, 16, 19]. Remission rate was recorded in all these five trials. The remission rate in MMF group was significantly higher than that in control group (Z = 2.48, *P* = 0.01) (Fig. 3a). When subgroup analysis for human race was taken, similar result was found in Asians but not in Caucasians or mixed races (Fig. 3a).

Reduction of urinary protein was also recorded in all the five trials. Of these five studies, four studies used 24 h urinary protein [12, 13, 15, 16] and one used urinary albumin to creatinine ratio [19]. Therefore, only four trials [12, 13, 15, 16] were included for analysis. The main analysis confessed that there were no significant differences in urinary protein reduction between the two groups (Fig. 3b). However, subgroup analysis for human race suggested that MMF monotherapy had a better efficacy on proteinuria alleviation compared to control in Asians (Z = 3.61, *P* = 0.0003) (Fig. 3b). There were three trials [13, 15, 16] reported serum creatinine doubling rate and progression to ESRD rate. The main analysis showed that there were no significant differences in serum creatinine doubling rate or progression to ESRD rate between the two groups (Fig. 3c-d). Moreover, similar results were found in subgroup analysis for human race (Fig. 3c-d).

Of these five studies, four studies [13, 15, 16, 19] reported adverse events, including infection, gastrointestinal symptoms, elevated liver enzymes, blood system changes, hair loss and irregular menstruation. Detail information was shown in Table 2. The main analysis showed that there were marginal differences in side effect rate between MMF group and placebo group in treating patients with IgAN (Z = 2.19, *P* = 0.03) (Fig. 3e). Additionally, subgroup analysis for human race suggested that similar results were found in Asians (Z = 2.08, *P* = 0.04) but not in Caucasians or mixed races (Fig. 3e).

**Table 2:** Adverse events reported in the included studies

Studies	Therapeutic regimen	Sample size	Infection	Gastrointestinal symptoms	Elevated liver enzymes	Blood system changes	Hair loss	Irregular menstruation	Total
Hogg 2015 [19]	MMF	23	0	2	0	0	0	0	2
	Placebo	27	0	1	0	0	0	0	1
Liu 2014 [18]	MMF + prednisone	42	2	0	0	0	0	0	2
	CTX + prednisone	42	2	3	1	1	1	3	11
Liu 2010 [17]	MMF + prednisone	20	0	0	1	0	0	0	1
	LEF + prednisone	20	0	1	1	0	0	0	2
Bao 2007	MMF + prednisone	19	2	2	0	1	0	0	5
	CTX + prednisone	18	3	4	1	2	2	2	13
Frich 2005	MMF	17	0	2	0	0	0	0	2
	Placebo	15	0	2	0	1	0	0	3
Tang 2005 [13]	MMF	20	3	2	0	2	0	0	7
	Placebo	20	0	0	0	0	0	0	0
Baes 2004	MMF	21	1	2	0	1	0	0	4
	Placebo	13	0	0	0	0	0	0	0

### Pooled results of studies using corticosteroid plus MMF therapy

Three studies evaluated the role of corticosteroid plus MMF therapy in treatment of IgAN patients [17, 18, 25] and all of these studies were from Asian. Remission rate was recorded in all the three trials. The pooled results of meta-analysis confessed that there were significant differences in remission rate between corticosteroid plus MMF regimen and other immunosuppressive agents plus corticosteroid regimen in treating patients with IgAN (Z = 2.49, *P* = 0.01) (Fig. 4a). Additionally, subgroup analysis for regimen suggested that compared to cyclophosphamide (CTX), MMF had a higher remission rate (Z = 3.14, *P* = 0.002) (Fig. 4a).

Reduction of urinary protein was also recorded in all the three trials. The pooled results of meta-analysis confessed that there were no significant differences in urinary protein reduction between corticosteroid plus MMF regimen and other immunosuppressive agents plus corticosteroid regimen in treating patients with IgAN (Fig. 4b). However, subgroup analysis for regimen suggested that MMF had a higher efficacy in urinary protein alleviation compared to CTX (Z = 5.42, *P* < 0.00001) (Fig. 4b). Two trials reported serum creatinine doubling rate [18, 25]. The pooled results of meta-analysis showed that compared to CTX, MMF had a lower serum creatinine doubling rate in treating patients with IgAN (Z = 2.01, *P* = 0.04) (Fig. 4c).

All the three studies reported adverse reactions, including infection, gastrointestinal symptoms, elevated liver enzymes, blood system changes, hair loss and irregular menstruation, detail information was shown in Table 2. The pooled result of meta-analysis showed on the basis of corticosteroid, MMF had a lower side effect rate than other immunosuppressive agents (Z = 3.72, *P* = 0.0002) (Fig. 4d). What’s more, in subgroup analysis for regimen, corticosteroid plus MMF regimen had a lower side effect rate than corticosteroid plus CTX regimen (Z = 3.74, *P* = 0.0002) (Fig. 4d).

### Publication bias

**Fig. 5:**
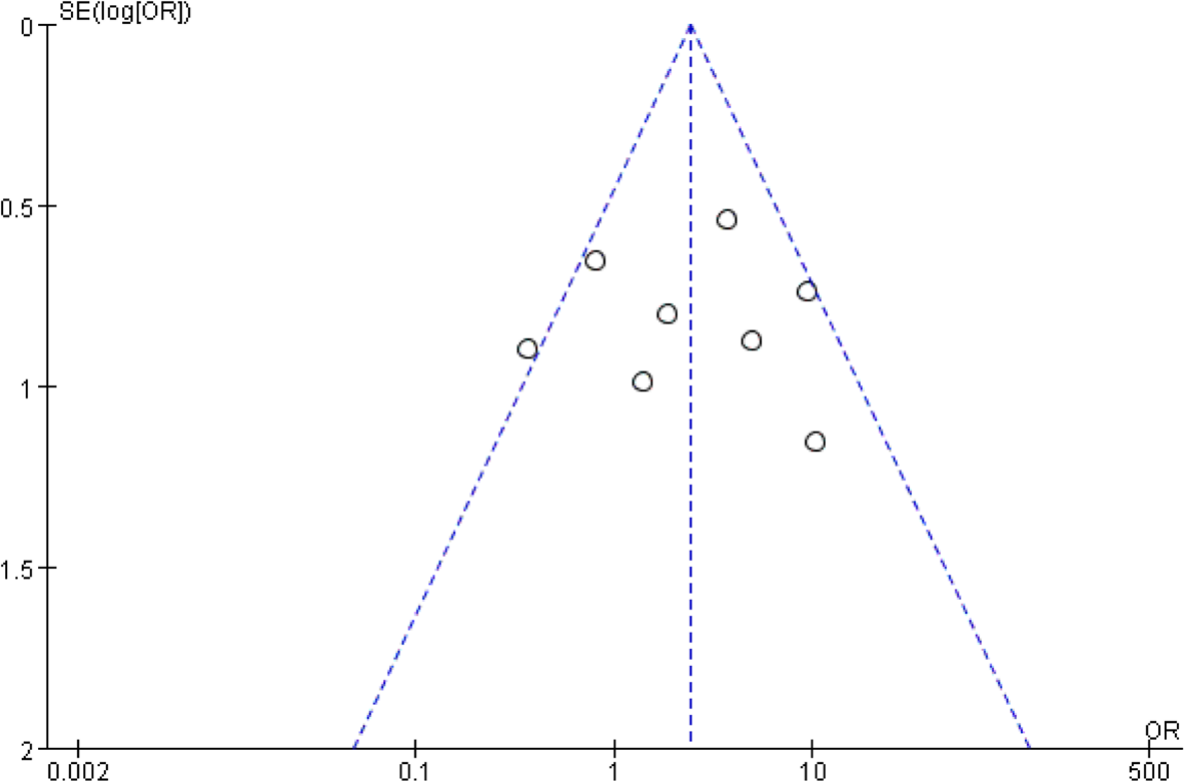
Funnel plot of included studies in this meta-analysis

Analysis of publication bias was conducted. No evidence of publication bias was found since the funnel plots was symmetrical based on a visual analysis (Fig. 5).

## Discussion

In a recent meta-analysis, the authors declared a relatively short course of MMF monotherapy might have favorable therapeutic effects such as remission rate and urinary protein reduction in IgAN [20]. In fact, it is indeed hard to explain why the outcomes from long course of MMF monotherapy are not better than that from short course of MMF monotherapy. In present study, we performed an update meta-analysis by adding one new RCT [19], and removing one RCT with unreliable data (in method part 19 and 14 participants were mentioned in MMF group and CTX group, respectively, but in result part 21 and 10 participants were mentioned in MMF group and CTX group, respectively) [21]. We made main analysis by including all the eight studies at first, and then we divided the studies into two categories, corticosteroid-free, MMF monotherapy [12, 13, 15, 16, 19] and corticosteroid plus MMF therapy [17, 18, 25].

For all the eight studies, our main analysis found higher remission rates in MMF group compared to control group. However, these results were not consistence with the previous meta-analysis [20]. Accumulating evidences have emerged and claimed that genetic factor contributes to the pathogeneses of IgAN [26, 27]. Furthermore, the genetic risk score is highest in Asians, intermediate in Caucasians, and lowest in Africans [28]. These genetic inconsistencies may explain why different human races have different responses to the same therapeutic regimen. Therefore, subgroup analysis by human race, which was not considered in previous meta-analysis [20], were performed in our study. Despite moderate to extreme heterogeneities were found in all comparisons, the heterogeneities were reduced or even disappeared in subgroup analysis, suggesting different human races may be one of the sources of heterogeneities in present meta-analysis.

In subgroup analysis by human race, two RCTs from Asians [12, 13] demonstrated obviously favorable outcomes, but two RCTs from Caucasians [15, 16] and one RCT from mixed races [19] failed to find significant differences between the two groups. Considering disease pathogenesis and the pharmacological effect of MMF, MMF is expected to play an important role in the treatment of IgAN. However, it was disappointed to see the negative results in Caucasians and mixed races from our present study. To be noticed, besides steroids and MMF, omega-3 fatty acid which was proven to be effective in treating IgAN [29] was also used in the RCT from mixed races [19]. Although all participants were administrated with omega-3 fatty acid during the entire trial, MMF treatment still could not significantly reduce proteinuria in patients with IgAN. The following reasons may explain the unexpected results. First, baseline proteinuria was severer in MMF monotherapy group than placebo group in one Caucasian RCT [15]. As both a major outcome and a positive predictor, the non-comparative baseline urinary protein might lead to a negative result. Second, a study design of 2:1 randomization was used in above Caucasian RCT to maximize the sample size [15]. Thus, the statistical power of this RCT was limited and we should interpret the result with caution. Third, in the other Caucasian RCT, baseline serum creatinine ranged from 2.2 mg/dl to 2.6 mg/dl [16]. Since patient selection, in other words baseline renal function directly influences the therapeutic effects of MMF [30], the advanced renal damage before MMF treating may cause non-favorable outcome. Fourth, all included five RCTs were single-center clinical trials, so the small sample size was another factor affecting the statistic power. Last but not least, corticosteroid recognized as the elementary medicine in treating IgAN [31] was used in only one of the five trials [12]. Maybe corticosteroid is basic and necessary for MMF to exert a therapeutic effect.

Immunosuppressive therapy like corticosteroid monotherapy and corticosteroid plus immunosuppressive agent therapy were well accepted in progressive IgAN treatment [32, 33]. However, which immunosuppressive agent is more effective is still unclear. Corticosteroid plus CTX therapy has been reported to obtain favorable outcome as a classic regimen in treating patients with IgAN [34, 35], while the adverse events caused by CTX can’t be ignored. Therefore, more safety immunosuppressive agents are needed. For corticosteroid plus MMF therapy studies, main analyses revealed better therapeutic effects in corticosteroid plus MMF group compared to corticosteroid plus other immunosuppressive agent group (CTX and LEF), including the remission rate and stable renal function. Our data were supported by previous studies [14, 36]. All the three RCTs were from Asians, so we made subgroup analysis by therapy regimen instead of human race. Despite high to extreme heterogeneities were found in some comparisons, the heterogeneities disappeared in subgroup analysis, suggesting different therapy regimens may be another source of heterogeneities in our meta-analysis.

In subgroup analysis by therapy regimen, compared to corticosteroid plus CTX but not LEF therapy, corticosteroid plus MMF therapy had a more superior effect on IgAN outcomes, such as remission rate, reduction of urinary protein and stable renal function. Compared to CTX, the better efficacy of MMF on the basis of corticosteroid was found not only in mild IgAN [25] but also in severe IgAN patients [18]. Our result was consistent with another retrospective study from China. In this study, the effects of MMF and CTX were compared in treating proliferative pathological IgAN. Data showed that combination of MMF and prednisone therapy lead to a beret renal survival compared to that of prednisone with CTX [37]. These results provided us evidence from evidence-based medicine. By removing one RCT with unreliable data [21], our new results were similar with the previous meta-analysis [20], suggesting the stability of this result. Both the efficacy and safety were comparative between corticosteroid plus MMF therapy and corticosteroid plus LEF therapy [17]. However, this conclusion was based on only one RCT, therefore, more RCTs evaluating corticosteroid plus LEF therapy are needed.

Besides human race and therapy regimen, renal histopathology was also an important factor affecting the efficacy of immunosuppressive therapy. For all the eight included studies, five studies had renal histologic assessment [13, 16, 18, 19, 25]. Although the degree of histologic damage at baseline between MMF group and control group was comparable, no study discussed the impact of renal pathology on therapeutic effects. In an observed study conducted by a Chinese group, the efficacy of MMF plus prednisone in treating Children with steroid-resistant IgAN was investigated. All biopsy samples were scored according to the Oxford classification. It was suggested that MMF plus prednisone therapy was effective in steroid-resistant children. However, unsatisfactory outcome was found in children with tubular atrophy/interstitial fibrosis [38].

Our main analysis of adverse events revealed that MMF monotherapy seemed to have a higher side effect rate because of the marginal difference between the two groups (*P* = 0.03). By restudying the included RCTs, we found only one RCT from Asians reported a significant higher side effect rate in MMF monotherapy group [13]. The main adverse events in MMF monotherapy group were gastrointestinal symptoms (8/81), infection (4/81) and blood system changes (3/81). Considering the marginal differences and small number of participants in this meta-analysis, to convince our result, more RCTs with big sample size are requested. In contrast to the above results, main analysis of adverse events showed that side effect rate in corticosteroid plus MMF group was much lower than that in corticosteroid plus other immunosuppressive agent group. And subgroup analysis by therapeutic regimen confirms the main analysis results. Except for infection, gastrointestinal symptoms and blood system changes, irregular menstruation (5/80), liver damage (3/80), blood system damages (3/80) and hair loss (3/80) in corticosteroid plus CTX regimen group were reported.

Our present meta-analysis has some limitations. First, not all included studies were high quality RCTs. Second, it will takes 15–30 years to progress to ESRD from IgAN onset [39]. Thus, with the follow-up period ranged from six to thirty-six months in these RCTs, it is difficult to observe obvious changes in kidney survival situation. Third, the majority of studies were from Asian patients, studies from other human races are needed.

## Conclusion

In summary, the evidences currently available show that IgAN patients in MMF group have a higher remission rate than that in control group, especially for Asians. In addition, MMF monotherapy offers benefits over placebo or corticosteroid monotherapy in treatment of patients with IgAN, but exerts more side effects. While MMF combined with corticosteroid regimen has a more efficacy and lower side effects compared with corticosteroid plus CTX regimen. Moreover, due to the methodological insufficiency more high quality RCTs with big sample size and from different human races are desired to obtain more rigorous and objective clinical evidence.

## Abbreviations

CI: Confidence interval
CTX: Cyclophosphamide
ESRD: End-stage renal disease
IgAN: Immunoglobulin A nephropathy
KDIGO: Kidney Disease Improving Global outcomes
MMF: Mycophenolate mofetil
OR: Pooled odds ratio
RCTs: Randomized controlled trails
WMD: Weighted mean difference
